# Research on improved black widow algorithm for medical image denoising

**DOI:** 10.1038/s41598-024-51803-3

**Published:** 2024-01-30

**Authors:** Hepeng Qu, Kun Liu, Lina Zhang

**Affiliations:** College of Information Technology, Jilin Agricultural University, Changchun, 130118 China

**Keywords:** Data processing, Image processing, Programming language

## Abstract

Improving the quality of medical images is crucial for accurate clinical diagnosis; however, medical images are often disrupted by various types of noise, posing challenges to the reliability and diagnostic accuracy of the images. This study aims to enhance the Black Widow optimization algorithm and apply it to the task of denoising medical images to improve both the quality of medical images and the accuracy of diagnostic results. By introducing Tent mapping, we refined the Black Widow optimization algorithm to better adapt to the complex features of medical images. The algorithm's denoising capabilities for various types of noise were enhanced through the combination of multiple filters, all without the need for training each time to achieve preset goals. Simulation results, based on processing a dataset containing 1588 images with Gaussian, salt-and-pepper, Poisson, and speckle noise, demonstrated a reduction in Mean Squared Error (MSE) by 0.439, an increase in Peak Signal-to-Noise Ratio (PSNR) by 4.315, an improvement in Structural Similarity Index (SSIM) by 0.132, an enhancement in Edge-to-Noise Ratio (ENL) by 0.402, and an increase in Edge Preservation Index (EPI) by 0.614. Simulation experiments verified that the proposed algorithm has a certain advantage in terms of computational efficiency. The improvement, incorporating Tent mapping and a combination of multiple filters, successfully elevated the performance of the Black Widow algorithm in medical image denoising, providing an effective solution for enhancing medical image quality and diagnostic accuracy.

## Introduction

With the widespread application of medical images in clinical diagnosis and research, ensuring image quality has become a critical factor in guaranteeing accurate diagnoses and reliable outcomes^1^. However, in practical clinical imaging, medical images are often subject to various noises originating from collection devices, the transmission process, as well as image storage and processing, leading to artifacts. The generation of these noises may result from factors such as equipment performance, interference in transmission channels, and limitations in processing algorithms. A comprehensive discussion of these real-world scenarios is necessary to better understand the sources and characteristics of noise.To enhance medical image quality, image denoising techniques have become a focal point of research^2^. Medical images may be affected by various types of noise under different circumstances. Gaussian noise typically arises from uncertainties in the internal electronic components of medical imaging devices, while salt-and-pepper noise may result from equipment aging or errors in the image transmission process, manifesting as random bright and dark pixels. Poisson noise is related to photon statistics and is common in radioactive isotope imaging, such as PET or SPECT^3^. Speckle noise may be caused by defects on the surface of imaging equipment or sensors, presenting as bright or dark spots in the image. However, traditional denoising methods face a trade-off between noise suppression and the preservation of image details, often leading to the loss of important details while maintaining clarity. Finding an efficient method that effectively denoises while preserving image details is a pressing challenge in the field of medical image research.

Significant progress has been made in the field of medical image denoising with deep learning. However, its reliance on large-scale annotated data, high computational resources, and model interpretability limits its widespread application in practical medical scenarios. Researchers actively address these challenges, striving to improve the reliability and practicality of deep learning in medical image denoising. Nevertheless, challenges persist in aspects such as generalization capability, parameter tuning, and data privacy. In recent years, optimization algorithms have emerged in the field of image denoising^4^. The Black Widow optimization algorithm, as an emerging optimization method, demonstrates outstanding capabilities in handling complex problems and global optimization, inspired by the unique hunting strategy of the black widow spider in nature^5^. However, its application in the field of medical image denoising is relatively limited, and there is insufficient research on parameter selection and algorithm convergence. In practical clinical imaging, noise occurrence may be influenced by factors such as equipment type, imaging mode, and processing workflow^6^. In-depth analysis of these factors will help better understand the characteristics and sources of noise in medical images, providing strong support for proposing rational image denoising techniques.

In this context, proposing an improved Black Widow optimization algorithm and applying it to the task of medical image denoising aims to effectively remove noise while maintaining image clarity and details. Combining Tent mapping with the Black Widow optimization algorithm (BWOA) in medical image denoising offers significant advantages. The introduction of Tent mapping enhances the algorithm's adaptability to the complex features of medical images, while the global search capability of the Black Widow optimization algorithm effectively addresses various noise environments. A diverse combination of filters, including Tent mapping, enables the algorithm to comprehensively handle different types of noise, improving the robustness of denoising effects. Through the optimization of parameters and iteration strategies, along with the nonlinear characteristics of Tent mapping, the algorithm not only improves convergence and efficiency but also better preserves the details of medical images. Extensive experiments and comparisons, covering common noise types and varying noise intensities in medical image datasets, demonstrate that the improved Black Widow optimization algorithm achieves significant performance enhancements in medical image denoising. Through this research, we aim to provide new insights and methods for the field of medical image denoising, further advancing the development and application of medical imaging.

## Research status

In the current research on medical image denoising, several prominent challenges are faced. Firstly, the generalization capability and interpretability of deep learning methods remain pressing issues^7^. Secondly, traditional approaches exhibit limitations in handling specific types of noise and complex medical scenarios. Additionally, the demand for large-scale datasets restricts the practical application of deep learning methods. Against this backdrop, addressing the balance between denoising effectiveness and computational efficiency, enhancing algorithm adaptability to different noise types and medical application scenarios, becomes an urgent problem in current research. The significance of medical images in modern healthcare is undeniable. However, their susceptibility to various noises poses significant challenges to clinical diagnosis and research. Current research on medical image denoising reflects three prominent trends. Firstly, deep learning methods, especially Convolutional Neural Networks (CNNs), have made significant progress, but their reliance on large annotated datasets and computational resources limits their widespread application. Secondly, traditional image processing methods, such as mean filtering and Gaussian filtering, remain the preferred choices for researchers, but there exists a trade-off between detail preservation and noise suppression. Thirdly, some studies aim to achieve medical image denoising in a data-driven manner by extracting information from the data itself. However, despite some progress, this field still faces challenges in terms of generalization capability, computational efficiency, and multimodal image denoising.

In specific studies, various medical image denoising algorithms have been proposed. For instance, Kumar designed an Optimal Vector Variational (ODVV) filter based on deep convolutional neural networks, utilizing the Feedback Artificial Lion (FAL) algorithm for execution^8^. Brzostowski introduced a new algorithm combining regularization techniques with variational mode decomposition to eliminate MRI data noise and enhance the quality of magnetic resonance scans^9^. Moser J focused on denoising through multi-echo (ME) data acquisition and noise reduction using Nordic, particularly for thermal noise elimination^10^. Nayak proposed a hybrid adaptive preprocessing algorithm, validating the impact of Gaussian noise on the reliability of medical image data^11^. Hellwig incorporated artificial intelligence (AI) and deep learning (DL) methods with a multi-layer neural network, integrating infrared technology into conventional PET imaging schemes^12^. den Boer applied a powerful semi-automatic depiction workflow for denoising diffusion-weighted magnetic resonance imaging, featuring a stack of Residual AGC Attention Blocks with short skip connections as a feature extractor for recovering underlying subtle details and textures in images^13^. Wang utilized a hybrid loss function composed of Weighted Patch Loss (WPLoss) and High-Frequency Information Loss (HFLoss), considering the inclusion of texture details in high-frequency information^14^. These algorithms demonstrate their respective advantages and adaptability in different medical application scenarios, providing diverse solutions for medical image denoising.However, despite the progress demonstrated by these solutions, there are some shortcomings. The lack of comprehensive comparisons between different methods makes it difficult to determine which approach is more effective in specific contexts. The neglect of different types of noise may limit the applicability of these methods in practical scenarios.

Some research focuses on addressing specific types of noise. For example, Yan Hongbo proposed a joint denoising algorithm based on two-dimensional variational mode decomposition (2D-VMD) and fast non-local means (FNLM) for speckle noise in medical ultrasound images^15^. Liu explored the impact of the Deep Learning Image Reconstruction (DLIR) algorithm on low-dose pelvic arterial CT angiography (CTA) images, introducing a new perspective^16^. Xinong proposed a threshold neighborhood mean algorithm effective in suppressing salt-and-pepper and Gaussian noise while preserving image detail information^17^. Wei introduced an adaptive Wiener filtering algorithm based on adaptive Wiener filtering and 2D-VMD for denoising sonar images^18^. Senyao proposed a shear speckle phase map denoising method based on an optimized particle swarm algorithm, improving local search capabilities through improved linear inertia weight adjustment and the introduction of a nonlinear weight assignment method^19^. Bingli introduced an adaptive image denoising technique using the lion pride optimization algorithm, aiming to simultaneously address multiple types of noise^20^. These studies provide more targeted solutions for handling specific noise scenarios. In the optimization algorithm employing Tent mapping, we selected the nonlinear characteristics of Tent mapping based on theoretical relevance to better capture the nonlinear noise structure in medical images. Considering the strong spatial correlation between adjacent pixels in medical images, we introduced Tent mapping to better utilize this information. The superior adaptability allows the algorithm to process noise to varying degrees based on the characteristics of different regions, better adapting to non-uniform noise distribution. By comprehensively addressing the characteristics of nonlinearity, spatial correlation, and adaptability, the Black Widow optimization algorithm with Tent mapping demonstrates stronger performance in medical image denoising. It improves adaptability to complex noise and structures while better preserving critical image details. This optimization strategy, through handling nonlinearity, spatial correlation, and adaptability, better meets the practical requirements of medical image denoising.

## Improved black widow optimization algorithm

### Overview of the proposed approach

**Figure 1:**
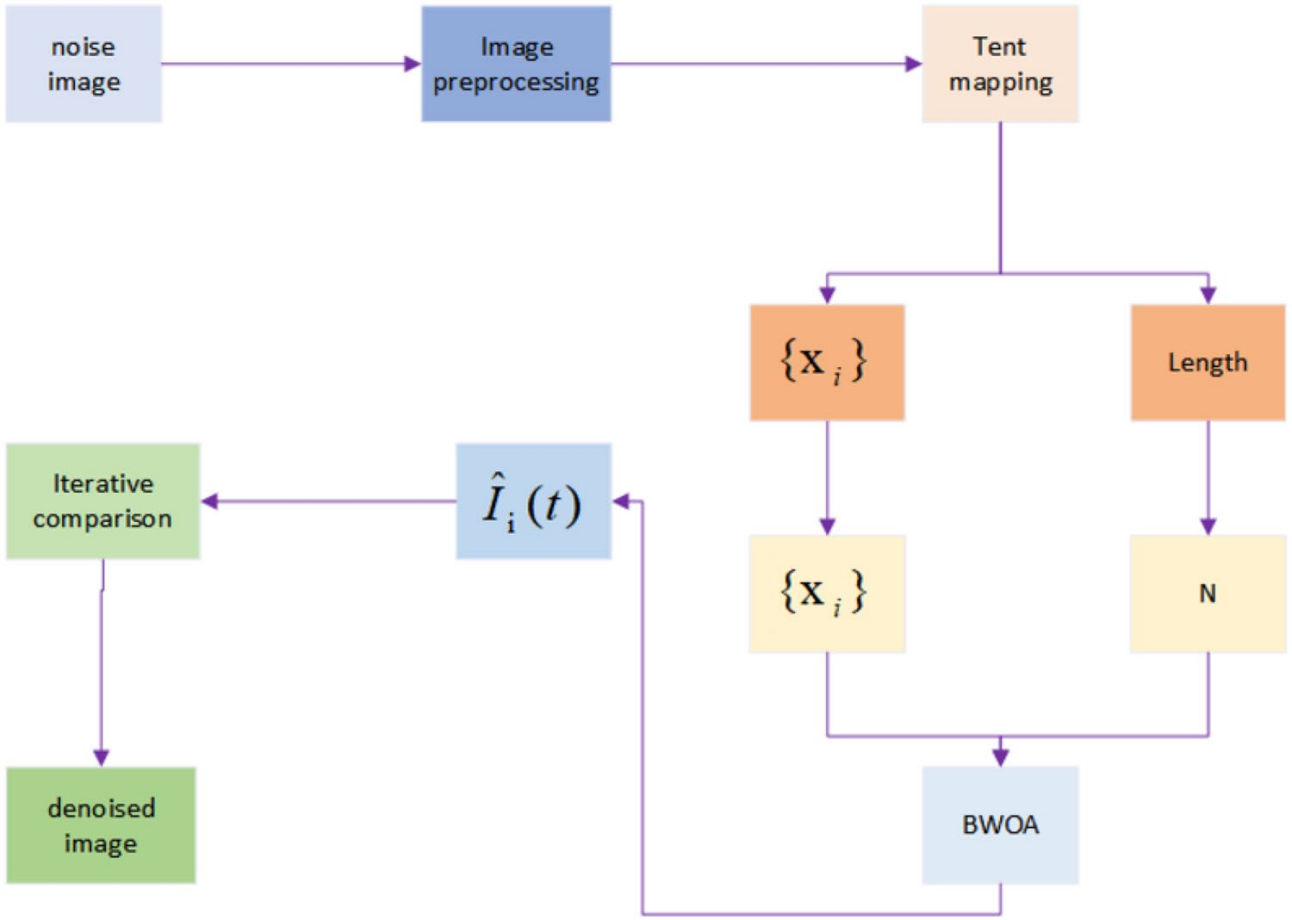
Medical image denoising process.

The process of improving the Black Widow optimization algorithm for medical image denoising with Tent mapping includes the following steps, as illustrated in Fig. 1. The first step involves inputting a medical image contaminated with noise and preprocessing the image. The second step utilizes Tent mapping to generate a chaotic sequence used to initialize the positions of the black widow individuals, thereby enhancing population diversity and global search capabilities. The third step employs the Black Widow optimization algorithm for image denoising. The movement strategy involves black widows adjusting their positions based on the current best image location and their own positions to balance the signal-to-noise ratio and structural similarity of the image. The pheromone mechanism updates the individual's pheromone values based on its fitness, reflecting its relative excellence. Higher pheromone values indicate superior individuals more likely to be followed by others. The replacement strategy is determined by the black widow's pheromone values, deciding whether to retain its position or replace it with the position of another individual to ensure diversity and stability. The fourth step yields an output of a denoised medical image with a higher peak signal-to-noise ratio (PSNR) and STRUCTURAL SIMILARITY INDEX (SSIM), preserving the image's edges and detail information.

### Principle of improved black widow optimization algorithm based on Tent mapping

**Figure 2:**
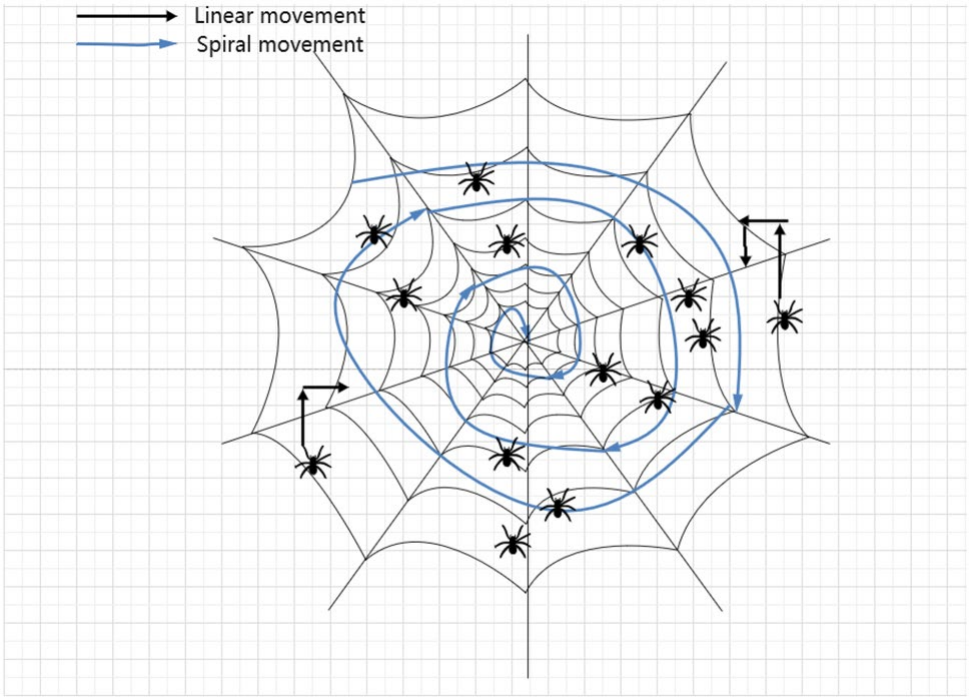
Schematic diagram of black widow optimization algorithm.

BWOA explores and exploits the search space by emulating various movement strategies observed in spiders during courtship, as depicted in Fig. 2. It primarily mathematically models different predation strategies of spiders, encompassing forced attacks, searching, and leaping towards prey. Additionally, a model representing the pheromone rate has been established^21^.

The steps of the improved black widow optimization algorithm based on Tent mapping are as follows: (1) Initialize the population of the improved black widow optimization algorithm, and set the algorithm parameters, such as population size, dimension, lower and upper bounds, number of iterations, etc. (2) For each population individual, use a combination of filters to denoise the image^22^. The filter combination can include non-local mean denoising, mean filter, median filter, bilateral filter, and Gaussian filter. First, a random vector is generated according to the Gaussian distribution, and then added to the current individual optimal position. Next, for each dimension in the individual, use Tent mapping to map the value of the dimension. Through this operation, the individual is updated and the updated value is guaranteed to be within the specified range. (3) Based on the denoised image quality evaluation index of each individual, use the improved black widow optimization algorithm to update and select individuals. The fitness value of each individual is calculated through the fitness function, and the optimal individual is selected based on the fitness value. (4) Iteratively execute steps 2 and 3 until the set maximum number of iterations is reached or the stopping criterion is reached. (5) Select the optimal individual to obtain the optimal filter parameter combination. (6) Use the optimal combination of filter parameters to denoise the original medical image. (7) Output the denoised medical image and save or perform subsequent analysis and processing. Tent chaos map is a nonlinear map that can generate sequences with good randomness and distribution^23^, which is used to initialize the position of the black widow. This can improve the diversity and global search capabilities of the population and avoid falling into local optimality. The formula is as follows:

1 $${{\text{x}}}_{{\text{i}}+1}=\left\{\genfrac{}{}{0pt}{}{ {\mathrm{\mu x}}_{{\text{i}}}, 0\le {{\text{x}}}_{{\text{i}}}\le \frac{1}{2} }{\upmu (1-{{\text{x}}}_{{\text{i}}}), \frac{1}{2}\le {{\text{x}}}_{{\text{i}}}\le 1}\right.$$

${{\text{x}}}_{{\text{i}}}$ represents the ith element in the chaotic sequence, while $\upmu$ is the control parameter, typically set to $\upmu =2$. The range of $\upmu$ can be adjusted to modulate the sensitivity and complexity of the chaotic sequence, thereby influencing the initial distribution of the black widow.

The initialization of the black widow's position involves mapping the sequence generated by the Tent chaotic mapping into the search space, obtaining the initial position of the black widow. This ensures that the black widow's position conforms to the range and precision of the search space. The mathematical formula for the initialization of the black widow's position is as follows:

2 $${{\text{x}}}_{{\text{i}}}(0)={{\text{x}}}_{{\text{min}}}+({{\text{x}}}_{{\text{max}}}-{{\text{x}}}_{{\text{min}}}){{\text{x}}}_{{\text{i}}+1}$$

In this context, ${{\text{x}}}_{{\text{i}}}(0)$ represents the position of the i-th black widow at the 0th iteration, while ${{\text{x}}}_{{\text{min}}}$ and ${{\text{x}}}_{{\text{max}}}$ denote the lower and upper bounds of the search space, and ${{\text{x}}}_{{\text{i}}+1}$ is the (i + 1)-th element generated by the Tent chaotic mapping.

The Black Widow Optimization Algorithm is an image processing algorithm that combines swarm intelligence and bio-inspired heuristics. It simulates the reproductive behavior of black widow spiders, optimizing the quality and visibility of images by continuously updating the positions of black widows. The Black Widow Optimization Algorithm primarily consists of three components: the movement strategy, information pheromone mechanism, and replacement strategy. The mathematical formula for the movement strategy is as follows:

3 $${{\text{x}}}_{{\text{i}}}({\text{t}}+1)=\left\{\begin{array}{c} {{\text{x}}}_{{\text{i}}}({\text{t}})-{\text{m}}{({\text{x}}}_{*}({\text{t}})-{{\text{x}}}_{{\text{i}}}({\text{t}})),\upbeta \ge 0\\ {{\text{x}}}_{{\text{i}}}({\text{t}})-{\text{m}}{({\text{x}}}_{*}({\text{t}})-{{\text{x}}}_{{\text{i}}}({\text{t}}))+\upbeta {({\text{x}}}_{*}({\text{t}})-{{\text{x}}}_{{\text{i}}}({\text{t}})),\upbeta <0\end{array}\right.$$

In the presented formula, ${{\text{x}}}_{{\text{i}}}({\text{t}}+1)$ corresponds to the updated position of the pixel point, while ${{\text{x}}}_{*}({\text{t}})$ represents the current optimal position of the pixel point. The variables m and $\upbeta$ introduce randomness into the algorithm, where m is a random number ranging from 0.4 to 0.9, and $\upbeta$ is a random number within the interval [− 1, 1]. The values of m and $\upbeta$ provide control over the black widow's movement speed and direction, influencing its search performance.

The Gaussian filter operates by computing the average pixel value within the filter window, and the coefficients of the filter window template decrease as the distance from the center pixel increases. The Gaussian filter expression is formulated accordingly.

4 $${\text{f}}({\text{x}},{\text{y}})=\frac{1}{{2\uppi }^{2}}{{\text{e}}}^{\frac{{{\text{x}}}^{2}+{{\text{y}}}^{2}}{{2\upsigma }^{2}}}$$

In the equation: f(x,y)represents the distance between pixels, and σdenotes the standard deviation of the Gaussian distribution.

Information pheromones play a crucial role in the mating process of spiders^24^. In the Black Widow Optimization Algorithm, spiders update their pheromone values based on their individual fitness, reflecting their relative quality. Higher pheromone values indicate superior individuals, making them more likely to be followed by other individuals. In this algorithm, the information pheromone rate of a black widow spider is defined as follows:

5 $${\mathrm{\varphi }}_{{\text{i}}}({\text{t}}+1)=\left\{\begin{array}{c}{\mathrm{\varphi }}_{{\text{i}}}(t)+\frac{{\text{f}}({{\text{x}}}_{{\text{i}}}({\text{t}}))}{{\sum }_{{\text{j}}=1}^{{\text{N}}}{\text{f}}({{\text{x}}}_{{\text{j}}}({\text{t}}))},f({{\text{x}}}_{{\text{i}}}(t))\ge f({{\text{x}}}_{{\text{i}}}(t+1))\\ {\mathrm{\varphi }}_{{\text{i}}}(t)-\frac{{\text{f}}({{\text{x}}}_{{\text{i}}}({\text{t}}))}{{\sum }_{{\text{j}}=1}^{{\text{N}}}{\text{f}}({{\text{x}}}_{{\text{j}}}({\text{t}}))},,f({{\text{x}}}_{{\text{i}}}(t))<f({{\text{x}}}_{{\text{i}}}(t+1))\end{array}\right.$$

where ${\mathrm{\varphi }}_{{\text{i}}}({\text{t}})$ represents the pheromone value of the ith individual at the tth iteration, ${\text{f}}({{\text{x}}}_{{\text{i}}}({\text{t}}))$ represents the fitness value of the ith individual at the tth iteration, and N is the population size.

When the pheromone value is less than or equal to 0.3, the individual will be replaced, and the position update formula is:

6 $${{\text{x}}}_{{\text{i}}}({\text{t}}+1)=\left\{\begin{array}{c}{\mathrm{ x}}_{{\text{i}}}(t+1),{\mathrm{ x}}_{{\text{i}}}(t+1)>0.3\\ {{\text{x}}}_{{\text{r}}1}(t)+\sigma ({{\text{x}}}_{{\text{r}}2}(t)-{{\text{x}}}_{{\text{r}}3}(t)), {{\text{x}}}_{{\text{i}}}(t+1)\le 0.3\end{array}\right.$$

where ${{\text{x}}}_{{\text{r}}1}$ and ${{\text{x}}}_{{\text{r}}2}$ represent two distinct individuals, and $\upsigma$ is either 0 or 1.

**Figure 3:**
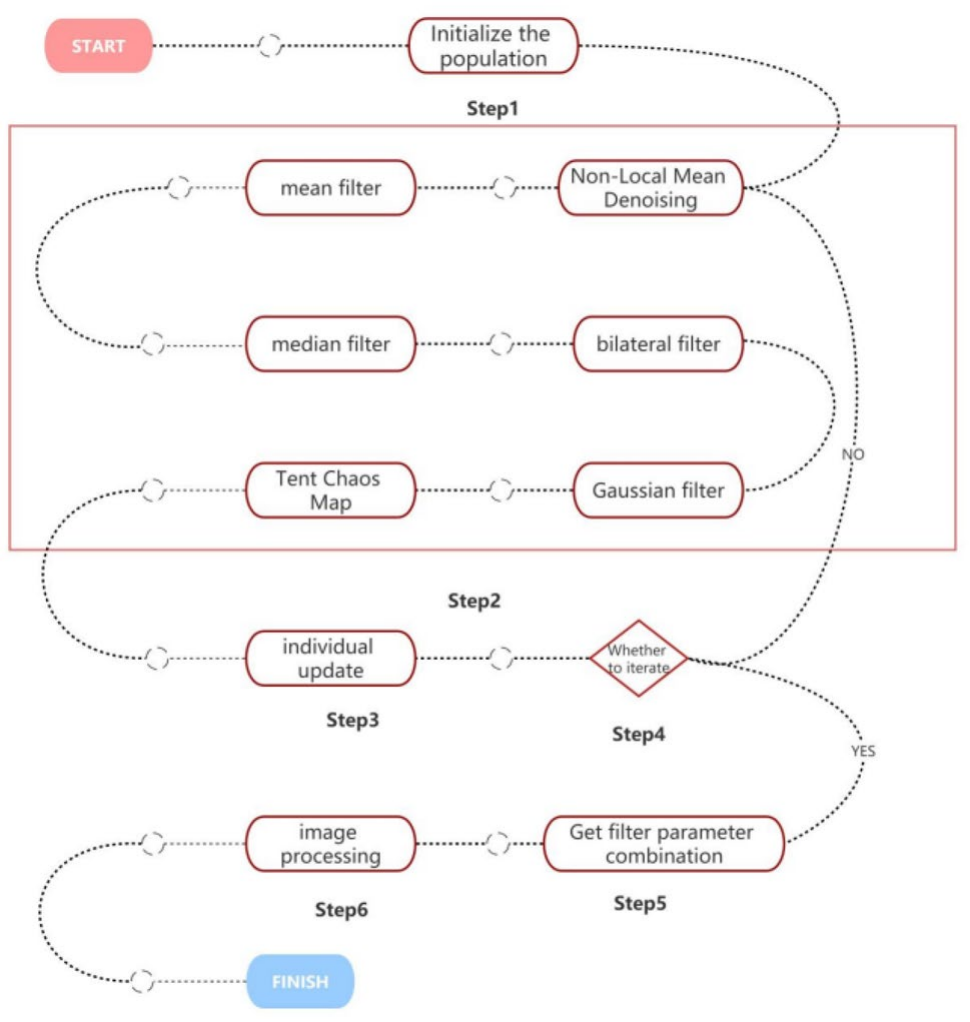
Flow chart of the algorithm.

In the Tent mapping, an extended range for $\upmu$ is introduced to facilitate the adjustment of the sensitivity and complexity of the chaotic sequence. In the Black Widow Optimization Algorithm, the ranges of ${\text{m}}$ and $\upbeta$ are expanded to enable better control over the spider's movement speed and direction. In the objective function for medical image denoising, the original maximization problem is transformed into a minimization problem to align with the solving approach of the Black Widow Optimization Algorithm. Simultaneously, the original single-objective function is converted into a multi-objective function to allow for the simultaneous consideration of image signal-to-noise ratio and structural similarity. The workflow is illustrated in Fig. 3.

### Filter combination

In order to denoise medical images containing different noises. First, the image is smoothed using a Gaussian filter to reduce noise by computing a weighted average of the area around the pixel. Next, a median filter is applied to remove salt and pepper noise or other outliers by sorting the pixel values in the area around the pixel and selecting the median. The image is then further smoothed using an averaging filter, which reduces the effect of noise by averaging the area around the pixel. Bilateral filters are used to smooth images and preserve edge information, filtering by considering the spatial distance between pixels and the similarity between pixel values. Finally, the sharpening filter enhances the edges and details of the image by calculating the difference between the original pixel and surrounding pixels and adding the difference back to the original pixel. This combination of filters is designed to reduce noise while maintaining image clarity and detail, and provides the basis for better visualization and analysis of medical images. Specific filter parameters and sequence selection should be adjusted according to image characteristics and denoising requirements to obtain the best results. The formula is as follows:

The working principle of the Gaussian filter is to calculate the mean value of the pixels in the filter window as the output value, and the template coefficient of the filter window decreases with the increase of the distance from the center pixel^25^. The Gaussian filter expression formula is as follows.

7 $${\text{f}}({\text{x}},{\text{y}})=\frac{1}{{2\uppi }^{2}}{{\text{e}}}^{\frac{{{\text{x}}}^{2}+{{\text{y}}}^{2}}{{2\upsigma }^{2}}}$$

In the formula, f(x,y) represents the distance between pixels; σ represents the standard deviation of Gaussian distribution.The median filter removes the noise in the signal without much impact on the signal itself^26^. Its principle is very simple: for a given window size, sort the values in the window, and then take the intermediate value as output. The formula is as follows.

8 $${\text{y}}[{\text{n}}]={\text{median}}({\text{x}}[{\text{n}}-{\text{k}}],..,{\text{x}}[{\text{n}}],...,{\text{x}}[{\text{n}}+{\text{k}}])$$

Among them, x is the original information of the window pixel value, y is the filtered window pixel value information, n is the current position, and k is the size of the picture pixel value.It is an indispensable operation in image preprocessing to suppress the noise of the target image while the mean filter retains the image details, and its processing effect will directly affect the effectiveness and reliability of subsequent image processing and analysis^27^. The formula is as follows.

9 $${\text{f}}({\text{x}},{\text{y}})=\sum_{{\text{m}},{\text{n}}}{\text{I}}({\text{x}}+{\text{M}},{\text{y}}+{\text{m}}){\text{K}}({\text{m}},{\text{n}})$$

where f(x, y) is the pixel output value, I(x + m, y + n) is the pixel input value, and K is the filter. The bilateral filter considers spatial information and gray similarity at the same time to achieve the purpose of edge preservation and denoising. It is simple, non-iterative, and local^28^. The advantage of the bilateral filter is that it can do edge preservation. The template weight ${\omega }_{r}$ determined by the difference of pixel values. The formula is as follows.

10 $$ \begin{gathered} {\upomega }_{{\text{r}}} \left( {{\text{i}},{\text{j}},{\text{k}},{\text{l}}} \right) = {\text{exp}}\left( { - \frac{{\left| {\left| {{\text{h}}\left( {{\text{i}},{\text{j}}} \right) - {\text{h}}\left( {{\text{k}},{\text{l}}} \right)} \right|} \right|}}{{2{\upsigma }_{{\text{r}}}^{2} }}} \right) \hfill \\ \omega_{d} \left( {i,j,k,l} \right) = exp\left( { - \frac{{\left( {i - k} \right)^{2} - \left( {j - l} \right)^{2} }}{{2\sigma_{r}^{2} }} - \frac{{\left| {\left| {h\left( {i,j} \right) - h\left( {k,l} \right)} \right|} \right|}}{{2\sigma_{r}^{2} }}} \right) \hfill \\ {\text{f}}\left( {{\text{i}},{\text{j}}} \right) = \frac{{\mathop \sum \nolimits_{{{\text{kl}}}} {\text{h}}\left( {{\text{k}},{\text{l}}} \right){\upomega }\left( {{\text{i}},{\text{j}},{\text{k}},{\text{l}}} \right)}}{{\mathop \sum \nolimits_{{{\text{kl}}}} {\upomega }\left( {{\text{i}},{\text{j}},{\text{k}},{\text{l}}} \right)}} \hfill \\ \end{gathered} $$

Among them, q(i,j) is the coordinates of other coefficients of the picture, h(i,j) indicates the pixel value of the image at point q(i,j); p(k,l) is the center coordinate point of the picture, and the corresponding The pixel value is h(k,l), and ${\sigma }_{r}$ is the standard deviation of the Gaussian kernel function on the pixel value range, which is used to control the weight of the pixel value. Generally, the weight value ${\omega }_{r}$ is called the value domain kernel, but whether it is the value domain kernel ${\omega }_{r}$ or the space domain kernel ${\omega }_{d}$, its size is between [0,1].

The sharpening filter can make the image through the first few filters look clearer and sharper, and enhance the details and features of the image^29^. In this paper, the sharpening kernel $3\times 3$ is used, and the formula is as follows.

11 $${\text{f}}({\text{x}},{\text{y}})=\sum_{{\text{s}}=-1}^{1}\sum_{{\text{t}}=-1}^{1}{\text{w}}({\text{s}},{\text{t}}){\text{g}}({\text{x}}+{\text{s}},{\text{y}}+{\text{t}})$$

g(x,y) is the input image and f(x,y) is the output filtered image.

### Performance verification and statistical analysis

In this paper, five commonly used quantitative indicators are selected to evaluate the denoising effect of images, including mean square error (MSE), peak signal-to-noise ratio (PSNR), structural similarity (SSIM), equivalent view number (ENL) and edge preservation index (EPI).

The smaller the mean square error (MSE) of the denoising result and the greater the peak signal-to-noise ratio, the higher the similarity between the image and the original image, the better the image features are preserved, and the better the denoising performance, the formula is as follows:

12 $${\text{MSE}}=\frac{1}{{\text{wh}}}\sum_{{\text{i}}=1}^{{\text{w}}}\sum_{{\text{j}}=1}^{{\text{h}}}{||{\text{M}}({\text{i}},{\text{j}})-{\text{N}}({\text{i}},{\text{j}})||}^{2}$$

In the formula: w and h are the size of the image; M(i,j) is the pixel size of the original image coordinates (i,j) pixels; N(i,j) is the reconstructed image coordinates after denoising (i,j) pixel size in pixels.

13 $${\text{PSNR}}=10\times {{\text{log}}}_{10}\frac{{({2}^{{\text{n}}}-1)}^{2}}{{\text{MSE}}}$$

In the formula: n is the number of bits of each sampling value, and the sampling points in the image signal are represented by 8 bits, so n is taken as 8. The larger the calculated peak signal-to-noise ratio, the smaller the noise signal of the denoised image, and the better the denoising effect of the image. SSIM compares the texture difference between the denoised image and the groundtruth image from the similarity in terms of brightness (l), contrast (c) and structure (s), the formula is as follows:

14 $${\text{SSIM}}({\text{x}},{\text{y}})=\frac{(2{\upmu }_{{\text{x}}}{\upmu }_{{\text{y}}}+{{\text{c}}}_{1})(2{\upsigma }_{{\text{xy}}}+{{\text{c}}}_{2})}{({\upmu }_{{\text{x}}}^{2}+{\upmu }_{{\text{y}}}^{2}+{{\text{c}}}_{1})({\upsigma }_{{\text{x}}}^{2}+{\upsigma }_{{\text{y}}}^{2}+{{\text{c}}}_{2})}$$

where ${\mu }_{x}$ is the mean of ${\text{x}}$, ${\mu }_{y}$ is the mean of y, ${\sigma }_{x}^{2}$ is the variance of x, ${\sigma }_{xy}$ is the variance of y, and 6 is the covariance of x and y. ${c}_{1}={({k}_{1}L)}^{2}$ and ${c}_{2}={({k}_{2}L)}^{2}$ are constants for stability. L is the dynamic range of pixel values. ${k}_{1}=0.01$, ${k}_{2}=0.03$.

ENL is often used to measure the smoothness of uniform regions in denoised images. The ENL formula for the i-th region of interest (ROI) in the image is as follows:

15 $${{\text{ENL}}}_{{\text{i}}}=\frac{{\upmu }_{{\text{i}}}^{2}}{{\upsigma }_{{\text{i}}}^{2}}$$

In the formula: ${\mu }_{i}$ and ${\sigma }_{i}$ represent the mean and standard deviation of the i-th ROI in the image. In the experiments, three ROIs are selected to calculate the average ENL. EPI is a performance index that reflects the degree of edge detail preservation of denoised images. The EPI formula of the i-th ROI in the image is as follows:

16 $$ \begin{gathered} {\text{image}}_{{{\text{Original}}}} = \mathop \sum \limits_{{\text{W}}} \mathop \sum \limits_{{\text{H}}} \left| {{\text{I}}_{{\text{n}}} \left( {{\text{x}} + 1,{\text{y}}} \right) - {\text{I}}_{{\text{n}}} \left( {{\text{x}} - {\text{y}}} \right)} \right| \hfill \\ {\text{image}}_{{{\text{Denoised}}}} = \mathop \sum \limits_{{\text{W}}} \mathop \sum \limits_{{\text{H}}} \left| {{\text{I}}_{{\text{d}}} \left( {{\text{x}} + 1,{\text{y}}} \right) - {\text{I}}_{{\text{d}}} \left( {{\text{x}} - {\text{y}}} \right)} \right| \hfill \\ {\text{EPI}} = \frac{{{\text{image}}_{{{\text{Original}}}} }}{{{\text{image}}_{{{\text{Denoised}}}} }}. \hfill \\ \end{gathered} $$

In the formula: ${I}_{n}$ and ${I}_{d}$ represent the original noise image and the denoised image respectively. If computed on the whole image, this metric may not accurately reflect edge preservation, since the gradients in homogeneous regions will be smaller after denoising. Therefore, only ROIs near the image boundaries are calculated.

## Experimental results

Selected 1588 images from the lung CT image dataset "Draft" for testing, with image dimensions of 256 × 256. Chose representative images to evaluate the denoising performance of the algorithms BM3D^30^, FASTATV^31^, NL-Means^32^, Med-cDiff^33^, AGC^34^, and BWOA on medical images. The experiments were conducted on a Windows 11 Professional edition system with a 12th Gen Intel(R) Core(TM) i5-12400F processor running at 2.50 GHz (base frequency 2.50 GHz) and 32 GB of RAM. The program execution environment was Python 3.10.11.

### Parameter selection of the proposed algorithm

The parameter selection for medical image denoising refers to determining the various parameters in the Tent mapping-enhanced Black Widow Optimization Algorithm to achieve optimal denoising effects. The control parameter μ of the Tent mapping, determining the sensitivity and complexity of the chaotic sequence, was set to $\upmu =2$ in our experiments to generate better randomness and distribution. The population size N, influencing the algorithm's search space and diversity, was set to N = 20 to ensure convergence speed and accuracy. The maximum number of iterations T, determining the algorithm's termination condition, was set to T = 50 for effective convergence. The random parameters ${\text{m}}$ and $\upbeta$ in the movement strategy, determining the Black Widow's speed and direction, were set to ${\text{m}}\in [\mathrm{0.4,0.9}]$ and $\upbeta \in [-\mathrm{1,1}]$ to balance exploration and exploitation. The threshold ${\mathrm{\varphi }}_{{\text{th}}}$ in the information pheromone mechanism, deciding whether the Black Widow needs to replace its position, ${\mathrm{\varphi }}_{{\text{th}}}=0.3$ was set in our experiments to increase population diversity and stability. Considering the proposed algorithm, as the number of iterations increases, the computation time also increases. Therefore, to find the optimal balance between computational performance and cost, setting a lower number of iterations is reasonable.

### Complexity analysis

The BM3D algorithm effectively utilizes the non-local self-similarity of images to enhance denoising performance but comes with a high computational complexity. The improved NL-means algorithm enhances denoising effectiveness and reduces computational complexity by optimizing the selection of neighborhood blocks, similarity calculations, and weight assignments. The horizontally adaptive NL-means algorithm, leveraging horizontal motion features, adaptively adjusts the search and neighborhood windows, improving denoising effectiveness but showing suboptimal results on images without horizontal motion. The adaptive fuzzy-weighted NL-means algorithm, employing fuzzy set theory, adaptively adjusts window size, improving denoising effectiveness but exhibiting diminished performance on non-Gaussian noise. The Black Widow Optimization Algorithm, simulating the mating behavior of black widow spiders, enhances denoising and convergence speed but with high computational complexity. The use of Tent mapping in the improved Black Widow Optimization Algorithm, under conditions of lower complexity, increases image diversity and randomness, thereby enhancing denoising effectiveness and the ability to escape local optima. The computational complexity formulas for each algorithm are provided in Table 1.

**Table 1:** Computational complexity public table.

No	Name	Operation formula
1	BM3D	${\text{o}}({{\text{N}}}^{2}{{\text{K}}}^{2}{{\text{N}}}_{1}{{\text{N}}}_{2}{{\text{T}}}_{1}{{\text{T}}}_{2})$
2	FASTATV	${\text{o}}({{\text{N}}}^{2}{{\text{K}}}^{2}{{\text{N}}}_{1}{{\text{N}}}_{2})$
3	NL-Means	${\text{o}}({{\text{N}}}^{2}{\text{M}})$
4	BWOA	${\text{o}}({{\text{N}}}_{3}{\text{M}}+{{\text{N}}}_{4}{{\text{d}}}_{1})$
5	Med-cDiff	${\text{o}}({{\text{N}}}^{2}{{\text{G}}}^{2}{{\text{ST}}}_{3})$
6	AGC	${\text{o}}({{\text{N}}}^{2}{{\text{d}}}_{2}{\text{L}})$

N represents the image size, K is the block size, ${{\text{N}}}_{1}$ is the maximum number of matching blocks for each reference block, ${{\text{N}}}_{2}$ is the search range for each reference block, ${{\text{T}}}_{1}$ is the complexity of the two-dimensional transformation, ${{\text{T}}}_{2}$ is the complexity of the one-dimensional transformation, M is the size of the neighborhood. ${{\text{N}}}_{3}$ denotes the population size, ${{\text{N}}}_{4}$ is the number of iterations for Tent mapping, ${{\text{d}}}_{1}$ is the dimension of Tent mapping, G is the variance of the noise, S is the complexity of the neural network, ${{\text{T}}}_{3}$ is the number of diffusion steps, ${{\text{d}}}_{2}$ is the number of channels in the network, and L is the number of layers in the network.

### Speckle noise image experimental results

**Figure 4:**
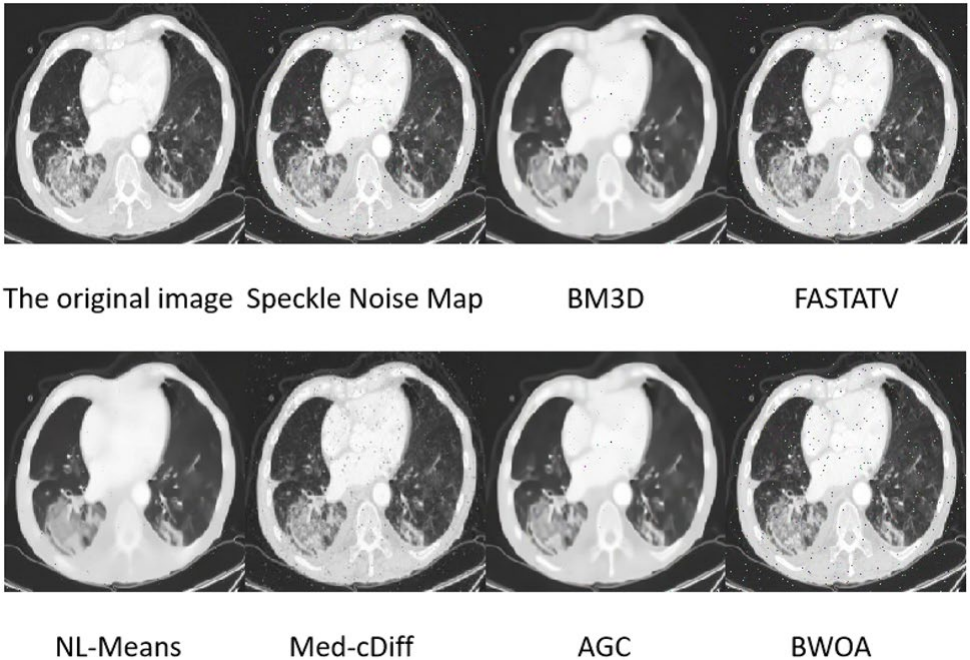
Experimental effect of speckle noise.

The experimental data comprised 397 lung CT images with speckle noise, and the results are illustrated in Fig. 4. The FASTATV algorithm exhibits limited capability in removing speckle noise, while both the BM3D and NL-Means algorithms show incomplete removal of speckle noise. The proposed improved algorithm, on the other hand, demonstrates superior visual effects in suppressing speckle noise while preserving edges and details. The performance data after smoothing speckle noise using different algorithms are presented in Table 2.

**Table 2:** Speckle noise performance table.

No	Name	MSE	PSNR	SSIM	ENL	EPI	Time
1	BM3D	0.917	27.430	0.790	1.509	0.729	5.623
2	FASTATV	0.804	28.303	0.844	1.386	0.202	3.710
3	NL-Means	0.627	29.401	0.778	1.476	0.711	2.859
4	BWOA	0.478	30.250	0.854	1.542	0.836	1.645
5	Med-cDiff	1.859	23.099	0.829	1.495	0.668	1.618
6	AGC	2.019	23.776	0.761	1.377	0.726	1.728

According to Table 2, it can be observed that our algorithm has certain advantages over the FASTATV, BM3D, NL-Means, Med-cDiff, and AGC algorithms in terms of removing image speckle noise. Through data comparison, our algorithm achieved a reduction of 0.439 in Mean Squared Error (MSE), an increase of 2.82 in Peak Signal-to-Noise Ratio (PSNR), an improvement of 0.076 in Structural Similarity Index (SSIM), an enhancement of 0.156 in Equivalent Number of Looks (ENL), and a boost of 0.614 in Edge Preservation Index (EPI). This indicates superior image quality, edge information, and structural similarity compared to other algorithms, with a comparable runtime to Med-cDiff and AGC algorithms.

### Poisson noise image experimental results

**Figure 5:**
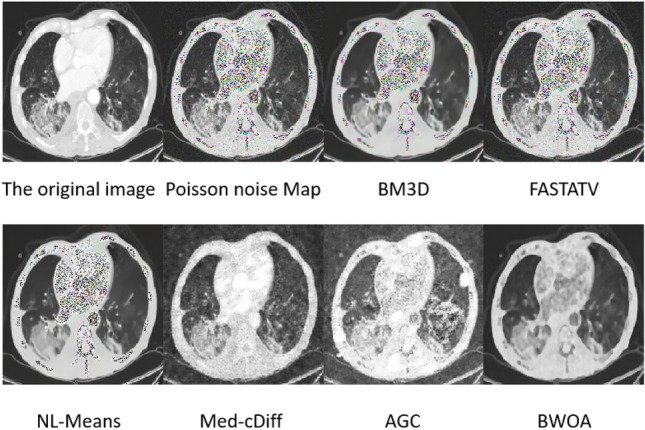
Effect diagram of Poisson noise experiment.

The experimental data consisted of 397 lung CT images with Poisson noise, and the result images are shown in Fig. 5. The effects of FASTATV, BM3D, and NL-Means algorithms on Poisson noise removal are similar, with BM3D outperforming FASTATV and NL-Means. Med-cDiff and AGC algorithms exhibit better Poisson noise removal than traditional methods but result in the loss of image edge details. The AGC algorithm enhances the brightness of the processed image. In comparison, our proposed improved algorithm, when suppressing Poisson noise in the image, clearly preserves the texture features compared to the original image, showing superiority over other algorithms in this aspect.

According to Table 3, our algorithm exhibits certain advantages over FASTATV, BM3D, NL-Mean, Med-cDiff, and AGC algorithms in removing Poisson noise from images. Through data comparison, our algorithm achieves a reduction of 0.276 in MSE, an increase of 1.898 in PSNR, a rise of 0.046 in SSIM, an enhancement of 0.263 in ENL, and an improvement of 0.138 in EPI. This indicates a good capability to preserve edge information, resulting in relatively low noise levels and overall superior image quality.

**Table 3:** Poisson noise performance table.

No	Name	MSE	PSNR	SSIM	ENL	EPI	Time
1	BM3D	0.761	18.140	0.615	1.652	0.658	5.285
2	FASTATV	0.835	17.259	0.585	1.436	0.729	4.170
3	NL-Means	0.700	17.481	0.608	1.516	0.691	2.635
4	BWOA	0.558	19.157	0.631	1.699	0.796	1.696
5	Med-cDiff	2.371	18.490	0.617	1.317	0.631	1.634
6	AGC	1.910	13.499	0.516	1.287	0.776	2.292

### Gaussian noise image experimental results

**Figure 6:**
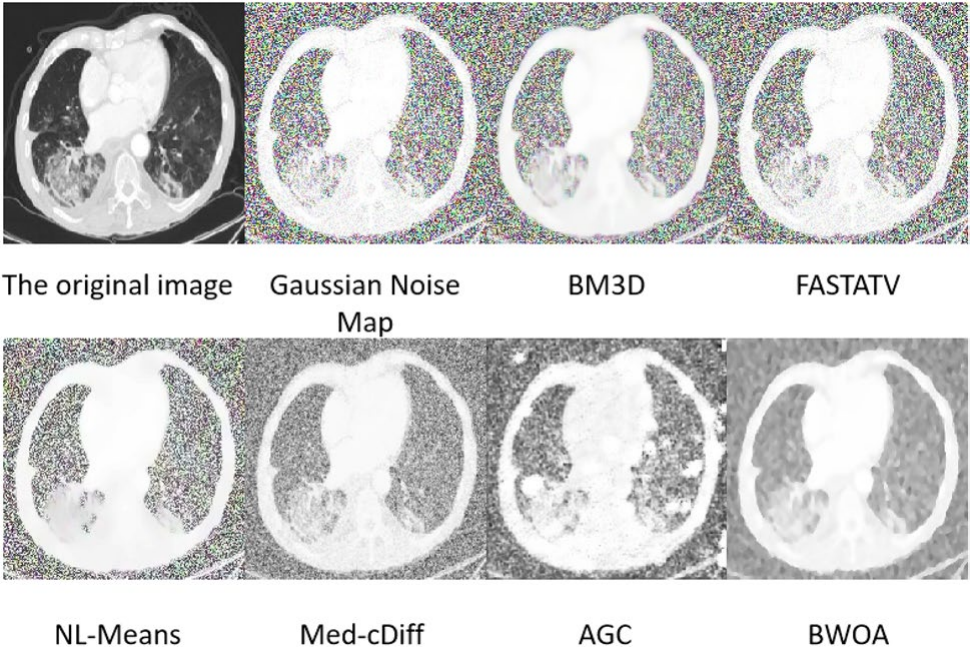
Experimental effect of Gaussian noise.

The experimental data consists of 397 lung CT images with added Gaussian noise, where the noise standard deviation is 25, as shown in Fig. 6. Neither FASTATV, BM3D, NL-Mean, Med-cDiff, AGC algorithms, nor the proposed improved algorithm effectively suppressed the Gaussian noise in the images. There is little difference in edge details and spatial structure among the images. While the denoising ability of the AGC algorithm surpasses traditional methods, its effectiveness against Gaussian noise is inferior to the algorithm proposed in this study. It is evident that our proposed algorithm exhibits a certain degree of suppression capability against Gaussian noise, with relatively preserved edge details in the images.

From Table 4, it is evident that our proposed algorithm exhibits certain advantages over FASTATV, BM3D, NL-Mean, Med-cDiff, and AGC algorithms in removing Gaussian noise from images. Through data comparison, our algorithm reduces MSE by 0.002, increases PSNR by 0.858, improves SSIM by 0.132, enhances ENL by 0.035, and raises EPI by 0.1. The denoising capabilities of the algorithm used in this study are comparable across Gaussian noise images, but the visual results are superior to other algorithms.

**Table 4:** Gaussian noise performance table.

No	Name	MSE	PSNR	SSIM	ENL	EPI	Time
1	BM3D	0.102	10.407	0.399	0.783	1.407	5.273
2	FASTATV	0.101	10.030	0.342	0.764	1.432	3.824
3	NL-Means	0.102	9.982	0.369	0.790	1.395	3.511
4	BWOA	0.100	10.840	0.474	0.799	1.435	1.798
5	Med-cDiff	1.173	10.828	0.411	0.758	1.329	1.668
6	AGC	1.110	8.382	0.341	0.714	1.399	3.684

### Salt and pepper noise image experimental results

**Figure 7:**
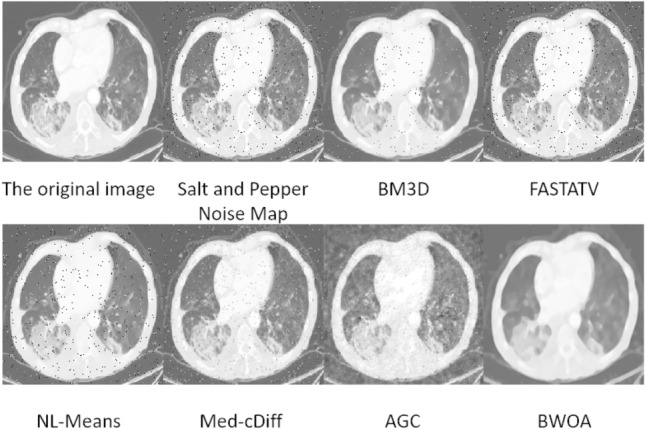
Experimental effect of salt and pepper noise.

The experimental data comprises 397 lung CT images with salt-and-pepper noise, and the results are illustrated in Fig. 7. FASTATV, BM3D, NL-Means, Med-cDiff, and AGC algorithms exhibit similar performance in removing salt-and-pepper noise; however, the resulting images still contain noticeable noise points. Med-cDiff algorithm mitigates the salt-and-pepper noise points, while AGC algorithm blurs the noise points, leading to the loss of image details. Our proposed algorithm demonstrates excellent performance in eliminating salt-and-pepper noise, although there is a risk of erroneously treating noise points as shadows in certain cases.

According to Table 5, the algorithm proposed in this study exhibits a certain advantage over FASTATV, BM3D, NL-Mean, Med-cDiff, and AGC algorithms in removing salt-and-pepper noise from images. By comparing the data, the proposed algorithm results in a reduction of 0.161 in Mean Squared Error (MSE), an increase of 4.315 in Peak Signal-to-Noise Ratio (PSNR), a boost of 0.105 in Structural Similarity Index (SSIM), a rise of 0.402 in Edge Number of Pixels (ENL), and an improvement of 0.598 in Edge Preservation Index (EPI). These findings indicate that the algorithm employed in this study enhances the structural similarity between processed images.

**Table 5:** Salt and pepper noise performance table.

No	Name	MSE	PSNR	SSIM	ENL	EPI	Time
1	BM3D	0.317	22.470	0.657	1.423	0.755	5.258
2	FASTATV	0.387	21.508	0.674	1.227	0.234	3.272
3	NL-Means	0.236	21.719	0.620	1.554	0.664	2.531
4	BWOA	0.226	25.823	0.725	1.629	0.832	1.765
5	Med-cDiff	0.593	25.207	0.705	1.288	0.776	1.649
6	AGC	0.498	23.121	0.674	1.212	0.824	1.982

### Ablation experiment

Using the same lung CT image dataset, the same image shows the impact of different algorithm improvements on the detection results, which are the black widow algorithm (BWOA), unsharp filter (BWOA1.0) and unsharp Tent denoising effect of filter (BWOA1.0) and chaos map (BWOA2.0) on lung CT images.

(1) Experimental results of speckle noise images

**Figure 8:**
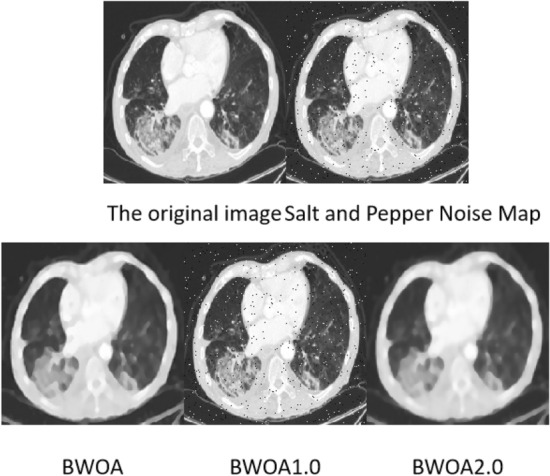
Speckle noise experimental effect diagram.

It can be seen from Fig. 8 that when BWOA1.0 and BWOA2.0 process images containing speckle noise, the denoising of the processed image is not complete. Compared with the original image, there are problems such as processing noise points into image shadows.

It can be seen from Table 6 that compared with BWOA1.0 and BWOA2.0, the optimization method adopted in this paper has certain advantages in terms of MSE, PSNR, SSIM and ENL performance, and is better than BWOA1.0 in terms of image edge details. Among them, since BWOA1.0 does not use Tent chaos mapping, the image noise level is much higher than the other two algorithms.

**Table 6:** Speckle noise performance table.

No	Name	MSE	PSNR	SSIM	ENL	EPI
1	BWOA	0.478	30.250	0.854	1.542	0.836
2	BWOA1.0	0.237	28.306	0.845	50.844	0.275
3	BWOA2.0	0.428	25.622	0.725	1.645	0.872

(2) Experimental results of Poisson noise images

**Figure 9:**
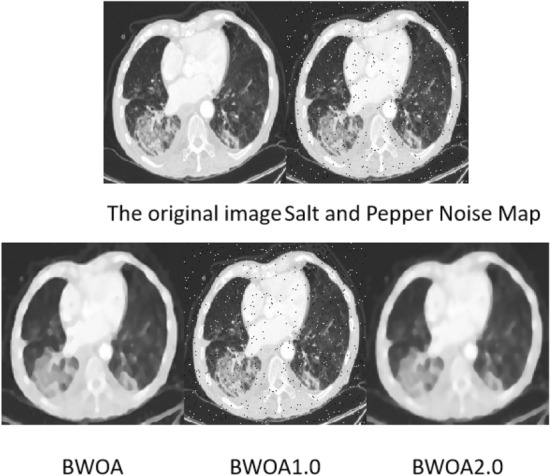
Poisson noise experimental effect diagram.

It can be seen from Fig. 9 that when processing images containing Poisson noise, none of the three algorithms can completely remove Poisson noise, and BWOA1.0 has the worst removal effect on Poisson noise. The difference in image texture between 0.0 and sex is not large.

As can be seen from Table 7, there is not much difference between BWOA and BWOA2.0. Both algorithms outperform BWOA1.0 in performance. Compared with BWOA and BWOA2.0, the processed image has lower noise level and higher image structure similarity. And it has better image edge preservation ability.

**Table 7:** Poisson noise performance table.

No	Name	MSE	PSNR	SSIM	ENL	EPI
1	BWOA	0.558	19.157	0.631	1.699	0.796
2	BWOA1.0	0.496	17.252	0.585	1.340	0.790
3	BWOA2.0	0.582	18.988	0.594	1.518	0.736

(3) Gaussian noise image experiment results

**Figure 10:**
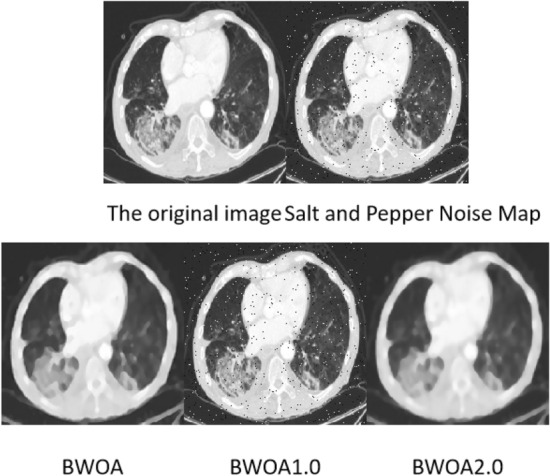
Gaussian noise experimental effect diagram.

It can be seen from Fig. 10 that among the three algorithms, BWOA1.0 has the worst ability to suppress Gaussian noise. BWOA and BWOA2.0 have the same ability to suppress Gaussian noise, but because BWOA uses a sharpening filter, the image processed by BWOA is better than the other two algorithms in terms of edge details.

It can be seen from Table 8 that the three algorithms deal with various performance indicators of images containing Gaussian noise, except for the mean value of the sum of squared differences. Average of the sum of squares of the difference between BWOA and BWOA1. These three algorithms are similar in removing Gaussian noise, where the Tent chaos map plays an important role.

**Table 8:** Gaussian noise performance table.

No	Name	MSE	PSNR	SSIM	ENL	EPI
1	BWOA	0.100	10.840	0.474	0.799	1.435
2	BWOA1.0	0.102	9.932	0.339	0.759	1.437
3	BWOA2.0	0.613	10.806	0.469	0.786	1.394

(4) Experimental results of salt and pepper noise images

**Figure 11:**
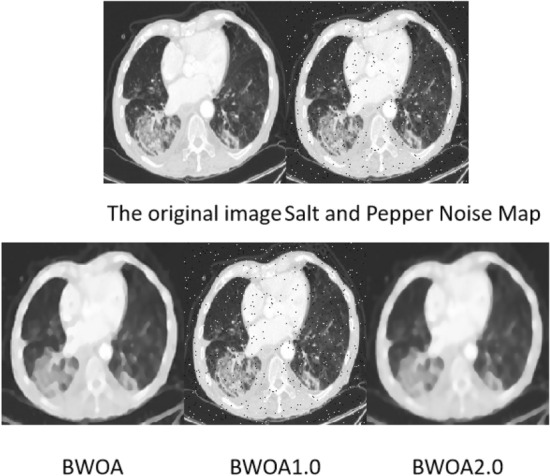
Effect diagram of salt and pepper noise experiment.

It can be seen from Fig. 11 that when BWOA1.0 processes an image containing salt and pepper noise, the denoising of the processed image is not complete. Compared with the original image, BWOA2.0 has problems such as processing noise points into image shadows. BWOA can better preserve image edge details.

It can be seen from Table 9 that compared with BWOA1.0 and BWOA2.0, the optimization method adopted in this paper has certain advantages in terms of MSE, PSNR, SSIM and ENL performance. As can be seen from PSNR, the image quality is higher than the other two algorithms. Among them, since BWOA1.0 does not use Tent chaos mapping, the image noise level is much higher than the other two algorithms. But BWOA is slightly inferior to BWOA2.0 in terms of image edge effects.

**Table 9:** Salt and pepper noise performance table.

No	Name	MSE	PSNR	SSIM	ENL	EPI
1	BWOA	0.226	25.823	0.725	1.629	0.832
2	BWOA1.0	0.347	21.482	0.675	26.154	0.306
3	BWOA2.0	0.4514	25.314	0.711	1.301	0.876

## Discussion

This study aims to enhance the denoising effect of medical images by introducing Tent mapping to improve the Black Widow Optimization Algorithm. In comparative experiments, the enhanced algorithm exhibited significant improvements in metrics such as PSNR and SSIM compared to the original algorithm, highlighting the crucial role of Tent mapping in enhancing image clarity and preserving information. Relatively speaking, the proposed algorithm demonstrated some noise suppression capability across various images. Further analysis indicated that Tent mapping contributes to better capturing local features, especially excelling in preserving subtle structures. In comparison with traditional and state-of-the-art algorithms, the proposed algorithm outperformed in terms of accuracy and robustness. It consistently demonstrated higher accuracy and robustness across various metrics. However, the study has certain limitations, and future work should delve into complex medical image denoising problems and the field of multi-modal image processing.

## Conclusion

This study explored the combination of Tent mapping and the Black Widow Optimization Algorithm with various filters for medical image denoising. Experimental results demonstrated a significant effect of this combined approach in removing noise and enhancing image details in medical images. Tent mapping contributed to enhanced contrast and detail, significantly improving the visual effects of medical images. Simultaneously, the Black Widow Optimization Algorithm optimized the parameter combination of filters, further enhancing the denoising effect. The combination of various filters played a crucial role in noise removal and image smoothing, effectively enhancing the edges and details of images, thereby improving the resolution and clarity of medical images. Future work will focus on further researching and optimizing this filter combination method, planning to introduce more types of filters and advanced algorithms to adapt to more complex medical image scenarios. The study will also explore adaptive parameter adjustments to improve algorithm adaptability. Additionally, efforts will be directed toward optimizing the algorithm for real-time image processing and addressing the effectiveness of handling large-scale medical image datasets. Collaborations with medical professionals for in-depth clinical validation will be pursued, and emphasis will be given to improving the algorithm's interpretability and transparency, promoting its widespread application in the medical field. In summary, the proposed method of combining Tent mapping and the Black Widow Optimization Algorithm with various filters provides an efficient solution for medical image denoising, offering valuable insights for future research and applications.

## Data Availability

The datasets used and/or analysed during the current study are available from the corresponding author on reasonable request.
